# Case of Puerperal Convulsions

**Published:** 1851-10

**Authors:** George Thompson

**Affiliations:** Jefferson, Tenn.


*Case of Puerperal Convulsions.* By George Thompson, M. D., of Jefferson, Tenn.—On the 6th of April, 1850, Mrs. T., aged about twenty-five, and rather delicate, was taken in labor with her fourth child. Labor was natural and speedy. She was delivered at 10 o'clock, A. M., of a stout, healthy child. Placenta was expelled without difficulty, and with no unusual hemorrhage. The uterus contracted well. Complained of pain ot her head, and her face was slightly flushed. She stated that she was very subject to head-ache when well, and she thought the present pain would be relieved as soon as she could get some sleep. I left her at 12 o'clock, as I then though doing well. I was summoned to her at about 3 o'clock P. M.; learned she had been attacked shortly after I left her with a violent convulsion, which had returned at intervals of less than an hour. She had then had three paroxysms. I found her partially comatose, restless, can be aroused, and answers rationally when questioned; complains of severe pain of her head, face flushed, pulse 170, hard and vibrating; tongue much swollen, from having been severely lacerated with the teeth during the convulsions.

I opened a large orifice in her arm, which bled freely. When she had lost about twenty ounces of blood, as violent a paroxysm of convulsions as I had ever witnessed, came on, which lasted about five minutes, leaving her entirely insensible. I continued the bleeding, until I had taken half a gallon. Her pulse now became very rapid, irregular, and could be but indistinctly felt at the wrist, hough strong in the temporal and carotid arteries; extremities cold. I now poured cold water very freely on her head, applied sinapisms to her legs and hot bricks to her feet. Sensibility returned in half an hour. I then gave her thirty grains of calomel, and shortly afterwards one and a half grains of sulphate of morphine. At 9 o'clock, P. M., no convulsion since the bleeding; still complains of pain in her head; in other respects easy. I left her with instructions to pour cold water frequently on her head, and to keep the warm applications to her feet.

7th, 9 o'clock, A. M. Has had no return of convulsions; slept some in the night; complains still of pain of her head; pulse 120; calomel has not operated; ordered one ounce castor oil; continue cold water to the head.

5 o'clock, P. M. Pain of the head continues; pulse 120; skin hot on the head and chest; extremities cool; lochia in the usual quantity; calomel has not operated. Continue cold applications to the head; repeat the oil once every four hours, with enemata, until the bowels are freely operated on.

8th, 9 o'clock, A. M. Says she feels much better this morning; slept well last night; countenance looks cheerful; still complains of pain of her head, though not so severe; some febrile reaction; pulse 120; extremities warm. Bowels have been freely evacuated during the night, motions copious, and fœtid; ordered blister to the neck, a mixture of equal parts of wine of ipecac, spts. nitre, and paregoric, in teaspoonful doses, to be taken once every two hours; continue cold applications to her head.

I consider it unnecessary to give further details of this case. It progressed to complete recovery very rapidly; and Mrs. T. was able to attend to the usual duties of her family as early as after any previous labor.

This is the eleventh case of puerperal convulsions which I have treated since 1817, all of which have recovered speedily, without leaving the slightest unhealthy condition of the system. I am inclined to think this success unusual, and I attribute it entirely to the freedom and rapidity with which I abstract blood. My treatment in other respects does not differ from that usually adopted.

The first case of this alarming disease which I ever saw, was in 1818. I had been practicing medicine but a short time, and was a young man as well as a young doctor. The patient was an athletic young woman, in her first pregnancy. When I saw her she had been delivered, during the paroxysms of convulsion, of a still born child, of about the usual size, and at the full period. She had suffered between thirty and forty of as violent paroxysms as I ever witnessed; was entirely insensible, with stertorous, laboring respiration; she could swallow nothing, and except when convulsed, lay with no evidence of life except breathing and pulse. Taken altogether, it seemed as hopeless a case as could be imagined. Bleeding and external applications were my only resource.

I had her taken out of bed and laid on an open jointed floor. I opened a large orifice in each arm, and cut both temporal arteries, and had blood flowing freely from all at the same time, determined to bleed her until the convulsions ceased, or as long as blood would flow. How much she bled I have no means of judging, for I designedly prevented any attempt to catch the blood, and the convulsions were so violent and so frequent, it could not have been caught if attempted. I suffered her to bleed until her pulse could not be felt at the wrist, and beat but feebly in the carotid arteries, by which time the convulsions ceased. Cold applications were made to the head, and blisters and sinapisms applied to the extremities. By the next day, sensibility had so far returned, that she could take medicine. The after treatment did not differ from that usually adopted in such cases. This woman recovered rapidly, without subsequent inconvenience.

This, my first case of puerperal convulsions, taught me to treat others. Since that time I have never bled my patient less than sixty ounces at a single bleeding, from as many and as large orifices as would insure the speedy accomplishment of my object. In many cases, I have little doubt I have taken double that quantity; for I have generally tried to prevent the quantity being known, for fear the friends of my patient should become alarmed and interpose to stop me before I wished.

My practice has been to bleed as long as I thought the bleeding itself would not endanger life. I have not had occasion to bleed the same patient a second time. I prefer to cut the temporal arteries in bad cases. In one case, when I failed to get blood as freely as I wished from other sources, I opened the external jugular vein. These eleven cases have occurred at various times since 1818; have been generally in young women in their first confinement.

In but one case has the delivery of the child arrested the convulsions, and in that case the child was delivered while blood was flowing.

In one case convulsions came on during the eighth month, without symptoms of labor; were arrested; came on again at the full period with labor; were arrested before delivery, which was afterwards effected by turning. The patient recovered in the usual time without a bad symptom.

I have stated that I thought the success of my treatment of this disease unusual. In verification of this statement I shall merely remark, that Dr. Lee, in his Clinical Midwifery, has reported forty-eight cases, (two of which I should not have called puerperal convulsions, but rather hysteria,) of which fourteen proved fatal at first, and two afterwards died of disease of the brain, the result of lesion left by the first attack.

I deem the present a suitable occasion to call the attention of the Society to a remedy in phlegmasia dolens, which I have not seen recommended in any treatise on that disease. I mean a simple roller bandage, firmly but evenly applied to the whole limb, from the toes to the pelvis. Those who have not applied this remedy to this disease, will be astonished at the speedy and entire relief of pain and inflammation produced by it. Try it, gentlemen, and your patients will bless you for the relief from pain of the most torturing character produced by it; and, instead of the mortification of having your cases under treatment for two or three months, you will find them cured in less than as many weeks.

I, of course, wish to be understood as recommending the bandage in this disease, in place of other external applications only, and not to the exclusion of such constitutional treatment as each case may require.

I prefer to keep the bandage wet with cold water or spirits.—*Ibid.*

